# Simultaneous Evaluation of Distal Femoral and Talar Cartilage Thicknesses and Neuropathic Pain in Patients with Early Rheumatoid Arthritis: Is There Any Relationship with Other Domains of Health?

**DOI:** 10.5152/ArchRheumatol.2026.25177

**Published:** 2026-01-16

**Authors:** Hüseyin Kaplan, Gizem Cengiz, Rukiye Akay, Mehmet Köksal, Havva Talay Çalış

**Affiliations:** 1 Department of Rheumatology, Aksaray Training and Research Hospital, Aksaray, Türkiye; 2 Division of Rheumatology, Department of Physical Medicine and Rehabilitation, Erciyes University Faculty of Medicine, Kayseri, Türkiye; 3 Department of Physical Therapy and Rehabilitation, Kayseri City Hospital, University of Health Sciences Faculty of Medicine, Kayseri, Türkiye; 4 Department of Physical Therapy and Rehabilitation, Kahramanmaraş Provincial Health Directorate, Afşin State Hospital, Kahramanmaraş, Türkiye

**Keywords:** Cartilage thickness, disease activity, neuropathic pain, rheumatoid arthritis, ultrasonography

## Abstract

**Background/Aims:**

To evaluate the distal femoral cartilage thickness (FCT) and talar cartilage thickness (TCT) and the frequency of neuropathic pain (NeP) in patients with early rheumatoid arthritis (RA) and to investigate their relationships with each other and other domains of health.

**Materials and Methods:**

The study included 35 patients with RA, who had a maximum disease duration of up to 1 year and 35 healthy controls (HCs) with demographic characteristics similar to the patients. Bilateral FCT and TCT of all participants were measured using ultrasonography. Pain, disease activity, level of functioning, quality of life (QoL), and anxiety and depression were evaluated with appropriate scales/questionnaires. Additionally, the presence of NeP was assessed with the painDETECT questionnaire.

**Results:**

Distal FCT (central, medial, and lateral regions) and TCT in the right and left extremities were not different between patients with early RA and HCs. Cartilage thicknesses were largely similar between the active and inactive patient subgroups. Patients with RA exhibited a significantly higher frequency of NeP than HCs (17.2% vs. 0%) (*P* = .012). There was a weak positive correlation between some parameters measuring disease activity and unilateral and/or bilateral FCT/TCT values. Conversely, C-reactive protein showed a weak negative correlation with right extremity (medial and lateral) FCT. No clear relationship was observed between cartilage thickness and NeP, functional status, QoL, or anxiety and depression. In addition, NeP showed significant associations with pain, most disease activity scores, anxiety, and QoL; however, it was not associated with swollen joint counts, acute phase reactants, or functional status.

**Conclusion:**

The findings indicate that distal FCT and TCT may not be affected in the early stages of RA. However, there is an increase in the frequency of NeP compared to the HCs. The presence of NeP seems to be associated with disease activity, QoL, and mental health, but not with cartilage thickness.

**Main Points**

- In early rheumatoid arthritis, femoral cartilage thickness (FCT) and talar cartilage thickness (TCT) values were comparable to those of healthy controls (HCs).
- The FCT and TCT values were not substantially associated with functional status, quality of life (QoL), active and inactive disease categories, and NeP.
- An increased frequency of neuropathic pain (NeP) was observed in patients with early RA compared with HCs, independent of cartilage thickness.
- The NeP scores appeared to influence disease activity, QoL, and anxiety scores.

## Introduction

Rheumatoid arthritis (RA) is a systemic autoimmune disease characterized by chronic synovial membrane inflammation, which results in irreversible articular cartilage and juxta-articular bone destruction.^1^ Pro-inflammatory cytokines produced by various immune cells (B cells, T cells, macrophages, etc.) due to autoimmunity, which represents the initial step in RA pathogenesis, stimulate synovial fibroblasts and lead them to polarize into tissue-destructive subsets. These tissue-destructive synovial fibroblasts promote the destruction of bone and cartilage by stimulating osteoclasts and expressing metalloproteinases, respectively.^2^ The RA-relevant inflammatory stimuli increase catabolic processes in chondrocytes and decrease their viability and proliferation.^3^ In addition to their role as target cells in RA, chondrocytes act as inflammatory cells that directly or indirectly accelerate joint destruction by exhibiting various changes.^3^^,^^4^ Consequently, cartilage damage may contribute to synovitis activation and persistence in RA.^5^

The most pronounced damage to the joints in patients with RA occurs in the first 2 years, making early diagnosis of the disease and appropriate therapeutic strategies crucial for averting joint destruction.^5^ Although conventional radiography (CR) is an easily accessible, inexpensive, and widely used imaging method worldwide for initial evaluation, it is inadequate in showing the early findings of RA.^5^^,6^ Ultrasonography (US) and magnetic resonance imaging (MRI), which provide significantly improved soft tissue delineation over CR, are the 2 most commonly used methods for early RA detection. These 2 imaging modalities can depict RA in the incipient stage, allowing for earlier and more accurate assessment of the cartilage and other structural components of the joints.^5^^-^^7^ While MRI remains the gold standard for articular cartilage evaluation, the use of US has become an important alternative owing to advances in technology.^4^ Advantages of US compared to MRI, such as lower cost, easy accessibility, real-time imaging, and the ability to simultaneously evaluate multiple joints, are making it increasingly accepted by clinicians.^4^^,^^8^ The decrease in cartilage thickness in RA patients was detected in the later stages of the disease and it was also found to decrease in the first year, called early RA, compared to healthy controls (HCs).^4^^,^^9^

In addition to loss of cartilage thickness, an increased frequency of neuropathic pain (NeP) was reported in patients with RA.^10^^,11^ Nociceptive pain resulting from stimulation of peripheral nociceptors is a crucial feature of the disease; however, lesions in pain pathways may also lead to NeP.^12^^,13^ Prolonged nociceptive stimuli from joints, upregulation of nerve growth factor-beta, and cytokine-driven sensitization of peripheral nerves, including tumor necrosis factor alpha, interleukin (IL)-1, and IL-6, have been reported to lead to the development of NeP in RA.^14^ In NeP, some impulses cause pain without stimulation, resulting in pain that does not respond to standard analgesics and disease-modifying antirheumatic drugs. Therefore, recognizing NeP and determining its frequency in these patients is critical.^13^ Despite increasing data on the sonographic evaluation of joints and the frequency of NeP in RA, to the best of knowledge, these 2 conditions have not been evaluated in the same study before. Additionally, data for both conditions in early RA are insufficient.

Therefore, this study aimed to assess the distal femoral cartilage thickness (FCT) and talar cartilage thickness (TCT) and the frequency of NeP in patients with early RA and in HCs and investigate their relationships with each other and other health domains (pain, disease activity of RA, functional status, quality of life (QoL), and anxiety and depression).

## Materials and Methods

### Study Participants

This cross-sectional study comprised 35 patients with RA, aged ≥18 years, who met the American College of Rheumatology/European Alliance of Associations for Rheumatology RA classification criteria^15^ and who applied to the Erciyes University Gevher Nesibe Hospital Rheumatology outpatient clinic and the Kayseri City Hospital Physical Medicine and Rehabilitation outpatient clinic. Following meticulous screening for lower extremity complaints or structural disorders that could cause abnormalities in lower extremity load distribution, 35 volunteer participants who did not currently report any musculoskeletal complaints and were relatives of the patients were recruited for HCs. The demographic characteristics (sex, age, weight, and height) of all participants were noted. The exclusion criteria included the following: age <18 years; a history of trauma, surgery, or infection in lower extremity joints; and a diagnosis of any metabolic, endocrine, or chronic liver/kidney disease and other rheumatic diseases (except RA). Moreover, those who had received steroid injections into their knee or ankle joints within the last 3 months were excluded. Because the target population in the study was patients with early RA, the maximum disease duration from symptom onset was limited to 1 year. The study was reviewed and approved by the Erciyes University Clinical Research Ethics Committee (dated March 29, 2023; no. 2023/211) and was conducted according to the Declaration of Helsinki. Informed consent was obtained from all participants.

### Laboratory and Disease Activity Evaluation

The results of the patients’ routine biochemistry, hemogram, C-reactive protein (CRP), and erythrocyte sedimentation rate (ESR) from their last visits and the anti-cyclic citrullinated peptide and rheumatoid factor (RF) tests requested at the time of diagnosis were recorded. To assess disease activity, various measures were calculated, as previously described, including swollen joint count (SJC), tender joint count (TJC), visual analogue scale (VAS) for pain and fatigue, global assessment from both patient (PtGA) and physician (PGA) perspectives, the Disease Activity Score 28 (DAS28) using both CRP (DAS28-CRP) and ESR (DAS28-ESR), the Clinical Disease Activity Index (CDAI), and the Simplified Disease Activity Index (SDAI).^16^ Then, active (DAS28 > 3.2, CDAI > 10, and SDAI > 11) and inactive (low disease activity and remission) disease states were defined according to the literature.^17^

### Ultrasonographic Evaluation

**Figure 1.**
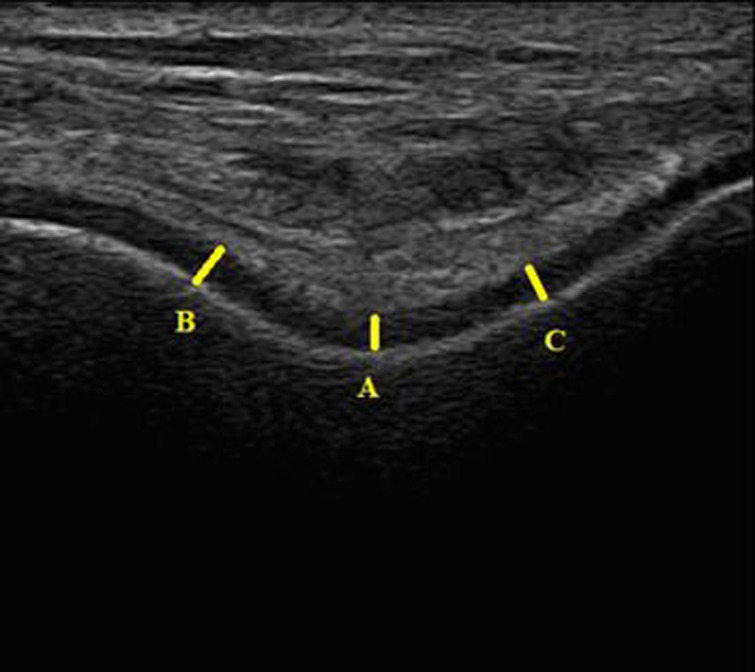
Ultrasonographic measurement of femoral cartilage thickness. A) Central (intercondylar), B) Medial, C) Lateral.

**Figure 2.**
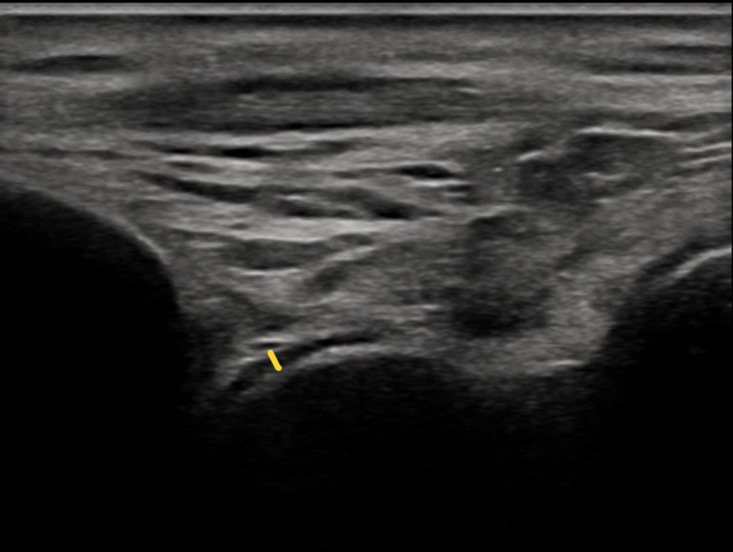
Ultrasonographic measurement of talar cartilage thickness.

Ultrasonographic measurements were performed by a physiatrist with over 5 years of practical experience, blinded to all clinical information of the patients. Ultrasonographic evaluation of bilateral FCT and TCT was conducted using a USG device (Philips ClearVue 550) with a 5-12 MHz linear probe. Bilateral distal FCT was measured in the axial plane and the suprapatellar region by placing the knee in maximum flexion, whereas bilateral TCT was obtained with the foot flat on the examination table in 90° flexion. While the distal FCT measurements were obtained from 3 regions (central [intercondylar], medial, and lateral) (Figure 1), the TCT was measured from a single region (Figure 2). Both knees and ankles were scanned for each participant. Each measurement was repeated 3 times by the same physician and the mean values were used for analysis.

### Scales/Questionnaires

For assessment of functional status, QoL, anxiety and depression, and NeP, the health assessment questionnaire (HAQ),^18^ RAQoL,^19^ Hospital Anxiety and Depression Scale (HADS-A/D),^20^ and painDETECT questionnaire (PD-Q)^21^ were administered as previously described. Based on PD-Q thresholds, patients scoring ≤12 are classified as unlikely to have a NeP component; scores of 13-18 indicate possible NeP; and scores ≥19 indicate probable NeP. All scales/questionnaires have validated Turkish versions.

### Statistical Analyses

The Shapiro–Wilk test evaluated whether the data followed a normal distribution. Summary statistics for continuous variables are presented as mean ± SD and those for categorical variables as numbers and percentages. Differences in normally distributed variables between the 2 independent groups were analyzed using the independent samples *t*-test, and the Mann–Whitney *U*-test was employed for nonnormally distributed variables. Chi-square tests were applied to compare categorical variables. The correlation between FCT, TCT, patient (clinical and demographic) characteristics, and scores of scales evaluating QoL, anxiety, and depression, and NeP was evaluated with the Spearman correlation analysis. Statistical Package for the Social Sciences Windows version 26.0 (IBM SPSS Corp.; Armonk, NY, USA) was used for data entry and subsequent statistical analyses. A *P *value of <.05 was considered statistically significant.

## Results

Table 1 summarizes the characteristics of patients with RA and HCs. No significant differences were detected between the 2 groups regarding sex, age, weight, height, and body mass index (BMI). In the patient group, the mean age was 53.00 ± 11.74 years and the mean disease duration was 4.03 ± 3.66 months.

The comparison of FCT and TCT values showed no significant difference between the patients and HCs (*P* > .05 for all). The frequency of NeP (both possible and likely) was significantly higher in the patient group than in HCs (*P* = .012) (Table 2). The mean PD-Q score in patients with RA was 7.97 ± 6.32.

Comparison of the FCT and TCT values and NeP frequency in active and inactive disease states (according to DAS28-CRP, CDAI, and SDAI scores) revealed that the left central FCT value was significantly higher in patients with active disease than in those with inactive disease (according to DAS28-CRP) (*P* = .033). Except for this variable, all other parameters were comparable between the groups (*P* > .05 for all) (Table 3).

Furthermore, the relationships between FCT and TCT values with demographic characteristics, clinical parameters, and scores of scales/questionnaires in patients with early RA were evaluated. A weak positive correlation was observed between TJC and unilateral extremity FCT (medial [*r* = 0.384, *P* = .023], central [*r* = 0.355, *P* = .036], and lateral [*r* = 0.362, *P* = .033]) and TCT (*r* = 0.347, *P* = .041) and between SJC and unilateral extremity FCT (*r* = 0.382, *P* = .023) and bilateral TCT (right [*r* = 0.473, *P* = .004] and left [*r* = 0.435, *P* = .009]). Additionally, a negative weak correlation was found between CRP and right extremity (medial [*r* = −0.364, *P* = .031] and lateral [*r* = −0.379, *P* = .025]) FCT. Conversely, a positive weak correlation was noted between disease activity scores (DAS28-CRP [*r* = 0.354, *P* = .037], CDAI [*r* = 0.365, *P* = .031], and SDAI [*r* = 0.340, *P* = .046]) and right extremity TCT. CDAI, SDAI, and RF titers showed weak positive correlations with medial (*r* = 0.397, *P* = .018; *r* = 0.359, *P* = .034; and *r* = 0.445, *P* = .007, respectively), central (*r* = 0.370, *P* = .029; *r* = 0.345, *P* = .042; and *r* = 0.365, *P* = .031, respectively), and lateral (*r* = 0.400, *P* = .017; *r* = 0.372, *P* = .028; and *r* = 0.363, *P* = .032, respectively) FCT values of the left extremity (Table 4). The relationship of NeP scores with demographic findings, clinical and laboratory data, disease activity parameters, and scores of assessment scales/questionnaires was also evaluated. In these analyses, weak positive correlations were observed between the PD-Q total scores and TJC (*r* = 0.363, *P* = .032), VAS-pain (*r* = 0.398, *P* = .018), VAS-fatigue (*r* = 0.431, *P* = .010), PtGA (*r* = 0.484, *P* = .003), PGA (*r* = 0.430, *P* = .010), DAS28-CRP (*r* = 0.430, *P* = .010), CDAI (*r* = 0.446, *P* = .007), SDAI (*r* = 0.485, *P* = .003), and RAQoL (*r* = 0.469, *P* = .004). PD-Q scores were moderately correlated with HADS-A and DAS28-ESR scores (*r* = 0.566, *P* = <.001 and *r* = 0.561, *P* = <.001, respectively). However, no significant correlation was found between age, BMI, disease duration, SJC, ESR, CRP, HAQ, and HADS-D scores and PD-Q scores (*P* > 005 for all).

## Discussion

This study revealed that FCT and TCT values were comparable to those of HCs in patients with early RA. The FCT and TCT values were not substantially associated with functional status, QoL, active and inactive diseases, and NeP. Additionally, some disease activity parameters (SJC/TJC, CDAI, and SDAI) and RF titers were positively correlated with unilateral FCT and/or TCT. However, the frequency of NeP was markedly higher among patients with RA compared with HCs (17.2% vs. 0%). The NeP scores showed significant associations with parameters of disease activity (except SJC, ESR, and CRP), QoL, and anxiety, but not with depression and functional status.

Recent insights into RA pathogenesis have elucidated the contributions of various immune cells (neutrophils, B cells, T cells, dendritic cells, monocytes/macrophages, etc.) and some non-immune cells (chondrocytes, fibroblasts, and osteoclasts).^22^ Chondrocytes originate from mesenchymal stem cells in the bone marrow and are the only cells found in cartilage tissue. In normal physiology, these cells are primarily responsible for synthesizing collagen and components of the extracellular matrix and are involved in the maintenance of the cartilage matrix and articular cartilage.^3^^,^^22^ Chondrocytes perform these functions in response to various cytokines and cellular signals. In inflammatory environments such as RA, chondrocytes become a target of inflammatory mediators and chondrocyte dysfunction occurs. In addition to being target cells, these activated chondrocytes contribute to increased cartilage damage, cytokines release into the synovium, and inflammatory and catabolic processes.^3^^,^^4^^,^^22^

According to the European Alliance of Associations for Rheumatology, “presence of early erosions” is one of the poor prognostic factors in RA disease management.^23^ Structural damage to joints can be detected using radiographs, and progression of damage can be evaluated with serial radiograph follow-ups. This method has advantages such as being easily accessible, relatively cheap, and reproducible, but it also has some limitations. It cannot directly visualize the various structures (e.g., soft tissues around the joint, synovium, cartilage, and bone marrow) on which the disease activity shows its effects and is inadequate in the early phase of the disease.^24^ Currently, MRI is the gold standard for evaluating articular cartilage in patients with RA.^4^ With more advanced molecular imaging tools including delayed gadolinium-enhanced MRI of cartilage, cartilage loss in early RA and its relationship with synovitis can be detected even before erosion develops.^25^ Musculoskeletal US is gaining more attention in the field of rheumatology, and its use is rapidly becoming widespread.^26^ The US has been shown to be beneficial in the diagnosis, follow-up, and prognosis of RA, as has MRI.^27^

Studies measuring cartilage thickness in patients with RA show that both small joints and large joints are considered, such as the wrist, hand (proximal interphalangeal [PIP] and metacarpophalangeal [MCP] joints), knee, ankle, and foot (metatarsophalangeal joint). In most of these studies, hand joints are addressed.^6^ Ogura et al^28^ showed that cartilage thickness in the PIP and MCP joints was thinner in patients with RA than in HCs; this thinning was significantly associated with disease duration, but not with disease activity. In another study evaluating the MCP joints, Hurnakova et al^29^ found that age and disease duration were linked to cartilage damage. Moreover, Mesci et al^9^ reported that only medial FCT thickness was decreased in patients with RA compared to HCs. However, they also noted that this decrease did not correlate with disease activity. In a cross-sectional study evaluating FCT and TCT in early RA, no correlation was found between FCT and TCT and disease duration, whereas a significant negative correlation was obtained with disease activity (DAS28) scores.^4^ In the current study, no clear relationship was observed between disease duration and presence of active disease and FCT and TCT. The lack of a significant relationship regarding disease duration may be due to the short disease duration in the RA cohort, as noted by Yıldırım et al.^4^ Regarding the effect of active disease, the results are consistent with those of Ogura et al^28^ and Mesci et al.^9^ The findings demonstrated different results regarding DAS28 scores and the presence of active disease compared to a study^4^ with similar methodology, which had found cartilage thickness to be associated with disease activity in early RA. These discrepancies may be due to differences in the time to reach the health center and the time to start treatment depending on the number of rheumatologists in the city where the study was conducted, differences in the patients’ compliance with treatment, and possible differences in the disease activities of the study patients. As previously noted in patients with osteoarthritis,^30^^,^^31^ cartilage swelling during the early stages of cartilage damage in early RA may have contributed to the inability to detect differences in FCT and TCT values between patients and HCs. A similar result was also obtained by Uysal et al^32^ in individuals with early-stage Parkinson’s disease. Comparison of active and inactive RA subgroups revealed that the left central FCT value was significantly higher in those with active disease than in those with inactive disease. This idea is supported by other cartilage thicknesses that were numerically, although not significantly, higher. According to previous studies, a negative association would be expected between CDAI/SDAI and some unilateral FCT and/or TCT values. However, the positive relationship observed in the study requires further investigation to determine whether this finding is driven by early cartilage swelling or other underlying factors.

Another point for discussion regarding the study results is the relationship between NeP—observed at a higher frequency in early RA—and cartilage thickness (FCT and TCT). In a study examining the frequency of NeP in patients with various rheumatic diseases, differing frequencies of NeP were found across disease subtypes; rates were significantly higher in osteoarthritis and ankylosing spondylitis than in the general population, but not in RA.^11^ Perrot et al^33^ reported the frequency of NeP in RA as 35.7%. Additionally, Ahmed et al^34^ noted a 33% frequency of NeP in patients with RA and a high positive correlation between NeP scores and VAS pain scores. Even in a relatively well-controlled RA cohort (approximately 75% of whom were in DAS28 remission), 38.4% of patients (17% likely NeP and 21.4% possible NeP) were observed to have features of NeP. Notably, these patients use higher rates of analgesics, have more TJC, and report more severe pain.^35^ In a recently published cross-sectional study, Büyük et al^36^ revealed that the frequency of NeP assessed with 3 different scales was significantly higher in RA patients than in HCs in all 3. Moreover, NeP scores were found to be positively correlated with VAS, RAQoL, and HAQ scores. In contrast to the reported prevalence of NeP of over 30% in RA cohorts with longer disease duration, the prevalence in the early RA cohort was 17.2%. The result was also lower than the frequency of NeP (29.6%) reported by Salaffi et al,^37^ who evaluated NeP in early RA. Although no significant association was detected, this appears to be the first study examining the relationship between NeP and cartilage thickness and functional status (assessed by HAQ) in patients with early RA, thereby contributing to the literature. The observed significant relationships between VAS pain and fatigue scores and PD-Q scores are consistent with previous findings.

Given the close association between NeP and pain, disease activity, and QoL scores, various RA disease activity measures that incorporate patient-reported subjective components are likely to yield higher scores in patients with NeP. The confounding effect of NeP can be decreased by using more objective tests. It is noteworthy that SJC, which is an important indicator of active disease in RA along with ESR and CRP^38^ (2 simple tests frequently used to assess disease activity in clinical practice), does not show a significant correlation with PD-Q scores. The presence of NeP should be carefully evaluated in patients with normal levels of acute phase reactants but high pain scores (VAS, TJC, PtGA, etc.) and consequently high disease activity scores. In addition to these associations, previous studies have demonstrated that PD-Q scores are related to depression, anxiety, and fatigue.^39^^,^^40^ Koop et al^35^ showed the significant effects of NeP on both physical and mental health in patients with RA with a median disease duration of 6 years. The relationship between NeP and anxiety found in early RA indicates that mental health has begun to deteriorate in the early disease period, even if functional status is not yet fully affected. Therefore, in patients with RA, NeP should be considered in evaluating disease activity and in assessing other health domains.

The most crucial strength of the study is that it evaluated both FCT and TCT and NeP simultaneously in the same cohort of patients with early RA. In addition, the inclusion of various questionnaires/scales that may be related to both makes it possible to address the issue from a more comprehensive perspective. However, the study had several limitations. First, the relatively small sample size and cross-sectional design limited the ability to draw causal inferences and precluded longitudinal follow-up to assess disease progression. Second, US findings were not confirmed by a different reference test such as MRI, and the relationship of cartilage thickness and NeP with US synovitis scoring systems was not evaluated. Third, the lack of assessment of intra- and inter-observer reliability represents another limitation related to US assessments. Fourth, although different scales are available for NeP detection, PD-Q was used. Different scales may reveal different NeP frequencies. Finally, as this was a single-center study, the findings may not be fully generalizable to broader populations.

This study showed no significant change in FCT and TCT in patients with early RA and that the frequency of NeP increased independently of cartilage thickness. The presence of NeP is associated with patient-reported pain, disease activity, QoL, and mental health. The NeP should be considered in patients under treatment who report high pain scores while acute phase reactants are at normal levels and there are no swollen joints. Further studies are warranted to understand when and how joint cartilage is affected in early RA, whether NeP development can be prevented, or, if present, how to diagnose and treat it in the most accurate way. It is anticipated that these results will stimulate additional research in this area.

## Figures and Tables

**Table 1.** Mean Values and SDs orNumber (%) of Demographic Characteristics, Clinical Parameters, and Assessment Scales/Questionnaires

	**Patients ** **(n = 35)**	**HCs** **(n = 35)**	*P*
Age, months	53.00 ± 11.74	53.00 ± 11.74	1.000*
Female sex, n (%)	23 (65.7)	23 (65.7)	1.000***
Weight, kg	77.11 ± 15.29	76.37 ± 12.56	.826*
Height, cm	163.54 ± 10.11	163.43 ± 7.71	.958*
BMI, kg/m^2^	29.10 ± 6.30	28.64 ± 4.53	.758*
Disease duration, months	4.03 ± 3.66	–	–
TJC	3.57 ± 3.32	–	–
SJC	0.71 ± 1.55	–	–
VAS-pain	5.51 ± 2.27	–	–
VAS-fatigue	6.46 ± 2.16	–	–
PtGA	5.34 ± 1.86	–	–
PGA	4.43 ± 1.56	–	–
ESR, mm/h	12.31 ± 9.32	–	–
CRP, mg/L	7.11 ± 9.38	–	–
DAS28-ESR	3.35 ± 1.03	–	–
DAS28-CRP	3.33 ± 1.00	–	–
CDAI	13.94 ± 6.80	–	–
SDAI	14.65 ± 6.99	–	–
RF titers, IU/mL	42.03 ± 51.25	–	–
Anti-CCP titers, U/mL	214.83 ± 212.67	–	–
HAQ	3.19 ± 5.01	0.00 ± 0.00	<.001**
RA-QoL	13.94 ± 9.72	–	–
HADS-A	7.94 ± 4.98	0.66 ± 0.77	<.001**
HADS-D	6.71 ± 4.29	0.51 ± 0.74	<.001**

Continuous variables are presented as mean ± SD and categorical variables are given in percentage. Bold emphasis in *P* values indicates statistically significant results.

*Student’s *t*-test.

**Mann–Whitney *U*-test.

***Chi-square test.

anti-CCP, anti-cyclic citrullinated peptide; BMI, body mass index; CDAI, Clinical Disease Activity Index; CRP, C-reactive protein; DAS, disease activity score; ESR, erythrocyte sedimentation rate; HADS-A, hospital anxiety and depression scale-anxiety; HADS-D, hospital anxiety and depression scale-depression; HCs, healthy controls; HAQ, health assessment questionnaire; PGA, physician global assessment; PtGA, patient global assessment; RAQoL, rheumatoid arthritis quality of life; RF, rheumatoid factor; SDAI, Simplified Disease Activity Index; SJC, swollen joint count; TJC, tender joint count; VAS, visual analogue scale.

**Table 2.** Comparison of FCT and TCT Values and NeP Frequency Between Patients and HCs

			**Patients ** **(n = 35)**	**HCs** **(n = 35)**	*P*
FCT, mm	Right extremity	Medial	1.98 ± 0.41	1.93 ± 0.37	.583*
		Central	1.99 ± 0.43	1.90 ± 0.45	.580**
		Lateral	1.97 ± 0.41	1.91 ± 0.35	.513*
	Left extremity	Medial	1.99 ± 0.34	1.91 ± 0.35	.290*
		Central	2.02 ± 0.34	2.11 ± 0.91	.514**
		Lateral	1.99 ± 0.35	1.93 ± 0.35	.461*
TCT, mm	Right extremity		1.29 ± 0.28	1.35 ± 0.25	.355*
	Left extremity		1.29 ± 0.29	1.35 ± 0.24	.289*
PD-Q subgroups, n (%)					.012***
≤12 (Unlikely NeP)			29 (82.8)	35 (100)	
13-18 (Possible NeP)			3 (8.6)	0 (0)	
≥19 (Likely NeP)			3 (8.6)	0 (0)	

Continuous variables are presented as mean ± SD and categorical variables are given in percentage. Bold emphasis in *P* values indicates statistically significant results.

*Student’s *t*-test.

**Mann–Whitney *U*-test.

***Chi-square test.

FCT, femoral cartilage thickness; HCs, healthy controls; NeP, neuropathic pain; PD-Q, painDETECT questionnaire; TCT, talar cartilage thickness.

**Table 3.** Comparison of FCT and TCT Values and NeP Frequency in Active and Inactive Disease Status According to Different Disease Activity Scores

			**DAS28-CRP**		*P*	**CDAI**		*P*	**SDAI**		*P*
			**≤3.2** **(n = 19)**	**>3.2** **(n = 16)**		**≤10** **(n = 12)**	**>10** **(n = 23)**		**≤11** **(n = 11)**	**>11** **(n = 24)**	
FCT, mm	R	Medial	1.95 ± 0.42	2.02 ± 0.41	.629*	1.83 ± 0.50	1.98 ± 0.39	.207*	1.83 ± 0.39	1.98 ± 0.39	.250*
		Central	1.94 ± 0.46	2.05 ± 0.39	.488*	1.84 ± 0.39	1.97 ± 0.45	.343*	1.83 ± 0.41	1.97 ± 0.45	.343*
		Lateral	1.93 ± 0.43	2.02 ± 0.39	.518*	1.81 ± 0.32	1.96 ± 0.39	.223*	1.83 ± 0.33	1.96 ± 0.38	.374**
	L	Medial	1.89 ± 0.33	2.11 ± 0.32	.070*	1.82 ± 0.33	1.98 ± 0.34	.136*	1.81 ± 0.34	1.98 ± 0.34	.142*
		Central	1.91 ± 0.32	2.16 ± 0.32	.033*	1.85 ± 0.30	2.11 ± 0.73	.120**	1.84 ± 0.32	2.11 ± 0.73	.115**
		Lateral	1.89 ± 0.35	2.10 ± 0.33	.076*	1.81 ± 0.33	1.99 ± 0.35	.134*	1.81 ± 0.35	1.98 ± 0.35	.127*
TCT, mm	R		1.21 ± 0.23	1.38 ± 0.32	.161**	1.20 ± 0.23	1.34 ± 0.27	.098**	1.21 ± 0.24	1.34 ± 0.27	.155*
	L		1.21 ± 0.23	1.38 ± 0.33	.189**	1.20 ± 0.23	1.35 ± 0.27	.087*	1.21 ± 0.24	1.34 ± 0.27	.135*
painDETECT group, n (%)					.525***			.261***			.079***
≤12 (Unlikely NeP)			17 (89.5)	12 (75)		11 (91.7)	18 (78.3)		11 (100)	18 (75)	
13-18 (Possible NeP)			1 (5.3)	2 (12.5)		1 (8.3)	2 (8.7)		0 (0)	3 (12.5)	
≥19 (Likely NeP)			1 (5.3)	2 (12.5)		0 (0)	3 (13)		0 (0)	3 (12.5)	

Continuous variables are presented as mean ± SD and categorical variables are given in percentage. Bold emphasis in *P* values indicates statistically significant results.

*Student’s *t*-test.

**Mann–Whitney *U*-test.

***Chi-square test.

CDAI, Clinical Disease Activity Index; DAS, disease activity score; FCT, femoral cartilage thickness; NeP, neuropathic pain; PD-Q, painDETECT questionnaire; SDAI, Simplified Disease Activity Index; TCT, talar cartilage thickness.

**Table 4.** Relationships Between FCT and TCT Values with Demographic Characteristics, Clinical Parameters, and Scores ofQuestionnaires/Scales in RA Patients

Variables	**FCT**						**TCT**	
	Right Extremity			Left Extremity			Right Extremity	Left Extremity
	Medial	Central	Lateral	Medial	Central	Lateral		
Age (years)	NS	NS	NS	NS	NS	NS	NS	NS
Weight	NS	NS	NS	NS	NS	NS	NS	NS
Height	NS	NS	NS	NS	NS	NS	NS	NS
BMI	NS	NS	NS	NS	NS	NS	NS	NS
Disease duration	NS	NS	NS	NS	NS	NS	NS	NS
TJC	NS	NS	NS	0.384*	0.355*	0.362*	0.347*	NS
SJC	NS	NS	NS	NS	NS	0.382*	0.473**	0.435**
VAS for pain	NS	NS	NS	NS	NS	NS	NS	NS
VAS for fatigue	NS	NS	NS	NS	NS	NS	NS	NS
PtGA	NS	NS	NS	NS	NS	NS	NS	NS
PGA	NS	NS	NS	NS	NS	NS	NS	NS
ESR	NS	NS	NS	NS	NS	NS	NS	NS
CRP	−0.364*	NS	−0.379*	NS	NS	NS	NS	NS
DAS28-ESR	NS	NS	NS	NS	NS	NS	NS	NS
DAS28-CRP	NS	NS	NS	NS	NS	NS	0.354*	NS
CDAI	NS	NS	NS	0.397*	0.370*	0.400*	0.365*	NS
SDAI	NS	NS	NS	0.359*	0.345*	0.372*	0.340*	NS
RF titers	NS	NS	NS	0.445**	0.365*	0.363*	NS	NS
Anti-CCP titers	NS	NS	NS	NS	NS	NS	NS	NS
PD-Q score	NS	NS	NS	NS	NS	NS	NS	NS
HAQ	NS	NS	NS	NS	NS	NS	NS	NS
RA-QoL	NS	NS	NS	NS	-0.338*	NS	NS	NS
HADS-A	NS	NS	NS	NS	NS	NS	NS	NS
HADS-D	NS	NS	NS	NS	NS	NS	NS	NS

*P < .05.

**P < .01.

anti-CCP, anti-cyclic citrullinated peptide; BMI, body mass index; CDAI, Clinical Disease Activity Index; CRP, C-reactive protein; DAS, disease activity score; ESR, erythrocyte sedimentation rate; FCT, femoral cartilage thickness; HADS-A, hospital anxiety and depression scale-anxiety; HADS-D, hospital anxiety and depression scale-depression; HAQ, health assessment questionnaire; NS, not significant; PGA, physician global assessment; PtGA, patient global assessment; RA, rheumatoid arthritis; RF, rheumatoid factor; SDAI, Simplified Disease Activity Index; SJC, swollen joint count; TCT, talar cartilage thickness; TJC, tender joint count; VAS, visual analogue scale.

## Data Availability

The data that support the findings of this study are available on request from the corresponding author.
